# *In Silico* Analysis of Glycosaminoglycan-Acemannan as a Scaffold Material on Alveolar Bone Healing

**DOI:** 10.1055/s-0041-1736592

**Published:** 2022-04-22

**Authors:** Sularsih Sularsih, Dian Mulawarmanti, Fitria Rahmitasari, Siswandono Siswodihardjo

**Affiliations:** 1 Department of Dental Materials, Faculty of Dentistry, Universitas Hang Tuah, Surabaya, Indonesia; 2 Department of Oral Biology, Faculty of Dentistry, Universitas Hang Tuah, Surabaya, Indonesia; 3 Department of Pharmaceutical Sciences, Faculty of Pharmacy, Universitas Airlangga, Surabaya, Indonesia

**Keywords:** glycosaminoglycan, acemannan, *in silico*, TLR-2 receptor

## Abstract

**Objective** This study aimed to analyze interaction between glycosaminoglycan-acemannan as a scaffold material and toll-like receptor-2 (TLR-2) receptor, which predicted the osteogenesis potency on alveolar bone healing ( *in silico* analysis).

**Materials and Methods** Docking interaction between glycosaminoglycan-acemannan and TLR-2 receptor using the *Molegro Virtual Docker* (MVD) program. The compounds of glycosaminoglycan-acemannan and TLR-2 receptor with the structure in the form of two- and three-dimensional images were analyzed, as well as the most stable structure. It was observed the interaction of the ligand on the cavity of the TLR-2 receptor structure. The energy required for the ligand and receptor interaction (Moldock score) was calculated with MPD program.

**Results** The chemical structure shows that glycosaminoglycan-acemannan is capable binding to the TLR-2 receptor with hydrogen bonds and strong steric interaction. The docking results were detected for five cavities where the compound binds to the TLR-2 receptor. The Moldock score of the ligand on the CAS-LYS-LEU-ARG-LYS-ILE-MSE[A] ligand was −95,58 Kcal/mol, that of acemannan was −91,96 Kcal/mol, and for glycosaminoglycan −61,14 Kcal/mol.

**Conclusion** The compound of glycosaminoglycan-acemannan as a scaffold material is able to bind with a TLR-2 target receptor, which predicted osteogenesis activity on alveolar bone healing supported by *in silico* analysis.

## Introduction

*In silico* study is a molecular modeling that can predict the physical and chemical characteristics of drug molecules, determines the description of compound–receptor interaction, and evaluates drug action at molecular levels through simulating the drug–receptor interaction process (docking). 1 2 *In silico* study is conducted to support the *in vivo* research and it could predict biological activity of a compound in drugs as anti-inflammatory or healing activity. 3 The interaction with a receptor has an important role in initiation of immune system to promote the bone healing process. 4

The membrane surface receptor which recognizes the substances in the cells of immune system is known as toll-like receptor-2 (TLR-2). Specific intracellular signaling is activated with the binding of ligands with TLR receptor. 5 The TLR-2 signaling pathway could identify polymer or botanical components in the alveolar bone healing process. The targeting of TLR receptor in the bone healing process has been a new challenge in some recent studies. 4 5

The osteogenesis process of alveolar bone by using a combination of *Aloe vera* and cancellous bovine xenograft increases TLR-2 expressions and osteoblast cells. 4 Several studies have reported that the acemannan compound of *A. vera* could support alveolar bone regeneration and periodontal regeneration therapy. The use of acemannan scaffold to the tooth socket can increase bone mesenchymal stem cells, vascular endothelial growth factor (VEGF), bone morphogenetic protein 2 (BMP-2), alkaline phosphatase expression, and mineralization. It is a natural candidate for bone regeneration biomaterial. A review in the *Journal of Functional Biomaterials* reported that *A. vera* is a natural plant which has high potential for application to tissue engineering scaffolds. 6 7 8 Glycosaminoglycan is the compound of polymer chitosan that can promote osteoblast formation. A chitosan and *A. vera* scaffold can decrease RANK expression and osteoclast resorption on alveolar bone healing. 9

Alveolar bone healing involves cellular and molecular processes, including the resorption and formation of soft and hard tissues. The injured periodontal ligament fibroblast will release endogen damage/danger-associated molecular pattern (DAMP) molecules that are captured by TLR-2 receptors then form a signal complex to activate macrophage cells. TLR-2 signal activates immune system, which plays a role in the bone healing. 4 10 This study aims to analyze interaction between glycosaminoglycan-acemannan as a scaffold material and TLR-2 receptor, which predicted the osteogenesis potency in alveolar bone healing ( *in silico* analysis).

## Materials and Methods

### *In Silico* Study

An *in silico* study was performed to simulate the drug–receptor interaction process (docking). The docking process of glycosaminoglycan-acemannan and TLR-2 receptor was analyzed using the software Molegro Virtual Docker (MVD). 1 2

### Creation of 2D and 3D Molecular Compounds

Two-dimensional (2D) and three-dimensional (3D) molecular compounds of glycosaminoglycan, acemannan, and CAS-LYS-LEU-ARG-LYS-ILE-MSE[A] ligand (PDB code: 1FYW) were downloaded from Research Collaboratory for structural Bioinformatics Protein Data Bank (RCSB-PDB). ChemBioOffice Ultra 12.0 program was (Cambridge Soft Co., Cambridge, United States) used to draw the 2D structure and to convert it into the 3D form. The most stable form of the stereochemical form of the compound was analyzed with MVD 5.5 program (CLC Bio, Aarhus, Denmark). 1 3 10 11

### Docking and Analysis of Amino Acids

Docking of interactions between glycosaminoglycan-acemannan and TLR-2 receptor structures used in the form of 3D images was performed with MVD program. 12 13 14 The docking process of (PDB code: 1 FYW) ligand and TLR-2 receptor were detected and find out the cavity binding to receptors. The 3D structures of glycosaminoglycan-acemannan in the fifth cavity were connected and analyzed the most stable binding to the TLR-2 receptor. The compound will automatically dock to the receptor and the energy required for the ligand and receptor interaction (Moldock score) was calculated with MPD program. 3 10 11

## Results

### *In Silico* Study Result

**Fig. 1:**
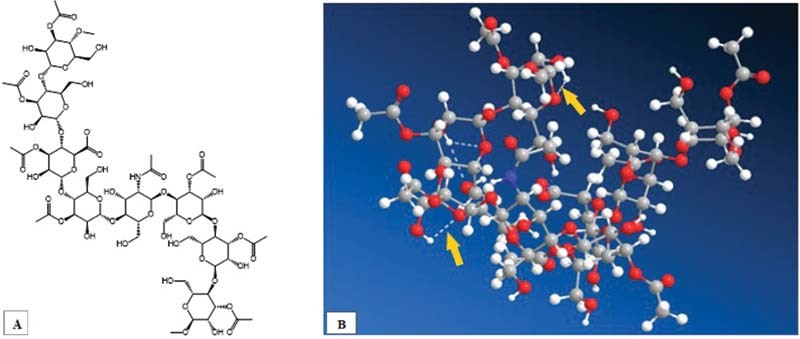
Two-dimensional images of acemannan ( **A** ), and three-dimensional images of acemannan (the arrows showing intramolecular hydrogen bond) ( **B** ).

**Fig. 2:**
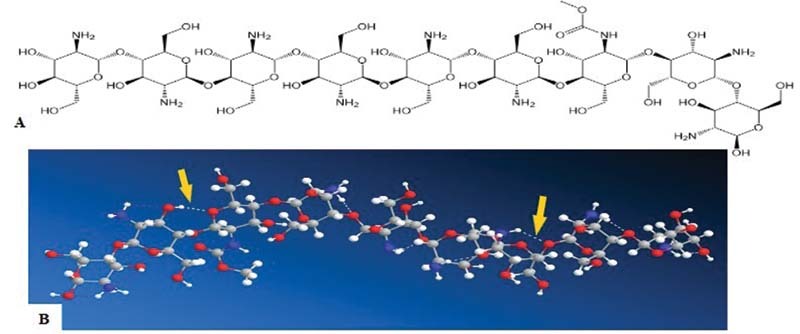
Two-dimensional images of glycosaminoglycan ( **A** ), and three-dimensional images of glycosaminoglycan (the arrows showing intramolecular hydrogen bond) ( **B** ).

The 2D and 3D images of glycosaminoglycan and acemannan are shown in Figs. 1 and 2 . There were intramolecular hydrogen bonds in 3D images of glycosaminoglycan and acemannan.

**Fig. 3:**
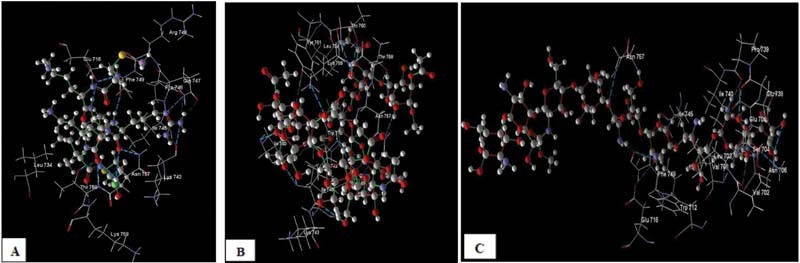
Three-dimensional images of interaction of CAS-LYS-LEU-ARG-LYS-ILE-MSE [A] ligand (PDB code: 1FYW) ( **A** ), acemannan ( **B** ), and glycosaminoglycan ( **C** ) with amino acids at TLR-2 receptor through hydrogen bonding.

*In silico* modeling results showed 3D images of interactions on the CAS-LYS-LEU-ARG-LYS-ILE-MSE [A] ligand (PDB code: 1FYW) of glycosaminoglycan-acemannan and amino acids at the TLR-2 receptor through hydrogen bonding and strong steric interaction. The CAS-LYS-LEU-ARG-LYS-ILE-MSE [A] ligand forms hydrogen bonds with the following amino acids: Lys 742, Pro 746, Gln 747, Thr 758, Asn 757, Lys 759, Glu 716, and Phe 749, and steric interaction with the following amino acids: Leu 734, Arg 748, Glu 716, Phe 749, lle 745, and Asn 757. Acemannan forms hydrogen bonds with the following amino acids: Lys 759, lle 740, Phe 749, Thr 760, Thr 758, Asn 757, and Lys 742, and steric interaction with the following amino acids: lle 745, Phe 749, Lys 742, Tyr 761, Lys 759, Lys 743, Thr760, Asn 757, Thr 758, and Leu 734. Glycosaminoglycan forms hydrogen bonds with the following amino acids: Val 708, Val 702, Ser 704, Asn 706, Glu 738, Pro 739, Glu 705, Glu 716, Asn 757, and Phe 749, and steric interaction with the following amino acids: Ser 704, Val 708, Asn 757, Phe 749, Trp 712, lle 740, lle 745, Glu 705, Leu 703, Glu 738, and Asn 706 ( Fig. 3 ).

**Fig. 4:**
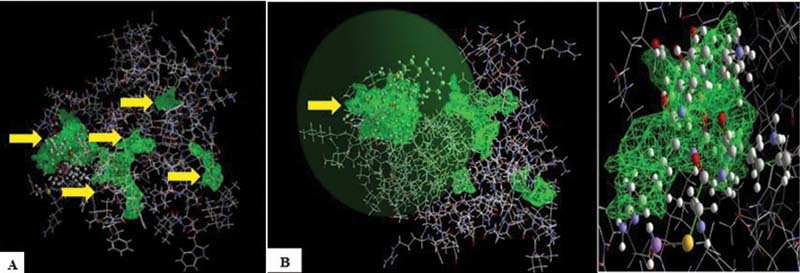
The images of five cavity results of docking process ligand and TLR-2 receptor. Marked *arrows* showing the cavity ( **A** ), cavity 1 was the most stable binding to TLR-2 receptor ( **B** ).

The results of docking process of the CAS-LYS-LEU-ARG-LYS-ILE-MSE [A] ligand (PDB code: 1FYW) and TLR-2 receptors were detected and there were five cavities where the compound binds to the TLR-2 receptor. Cavity 1 was the most stable binding to TLR-2 receptor ( Fig. 4 ).

The TLR-2 target amino acids with hydrogen bond and strong steric interaction and Moldock scores are shown in Table 1 . The amount of energy required for ligand and receptor interactions or the Moldock score of the ligand on the CAS-LYS-LEU-ARG-LYS-ILE-MSE [A] ligand was −94.34 Kcal/mol, for acemannan was −92,85 Kcal/mol, and for glycosaminoglycan was −62,19 Kcal/mol.

**Table 1:** The TLR-2 target amino acids with hydrogen bond and steric interaction and MolDock score

No	The compound	Amino acids with hydrogen bond	Amino acids with steric interaction	Mean MolDock score (Kcal/mol)
1	CAS-LYS-LEU-ARG-LYS-ILE-MSE [A] ligand	Lys 742, Pro 746, Gln 747, Thr 758, Asn 757, Lys 759, Glu 716, Phe 749	Leu 734, Arg 748, Glu 716, Phe 749, lle 745 and Asn 757	−94.34
2	Acemannan	Lys 759, lle 740, Phe 749, Thr 760, Thr 758, Asn 757, Lys 742	lle 745, Phe 749, Lys 742, Tyr 761, Lys 759, Lys 743, Thr760, Asn 757, Thr 758 and Leu 734.	−92.85
3	Glycosaminoglycan	Asn 757; Phe 749; Val 708; Val 702; Ser 704; Asn 706; Glu 738; Pro 739; Glu 705; Glu 716	Ser 704; Val 708; Asn 757; Phe 749; Trp 712; lle 740; lle 745; Glu 705; Leu 703; Glu 738; Asn 706	−62.19

## Discussion

The chemical structure of a drug determines its activity. Molecules of a drug that bind to a receptor or ligands may activate receptor and decrease or increase a particular cell function to lead cellular response. 12 The TLR-2 is membrane protein receptor regulating cellular biochemical processes. It recognizes pathogen-associated molecular patterns on microbial pathogen and stimulates cytokines for initiation of the immune system. The signaling marker derived from TLR-2 is known as the signaling pathway of botanical component. 4 5

The physical and chemical characteristics of drug molecules determine the description of compound and receptor interaction and can be predicted by an *in silico* study. The action of drugs at the molecular and atomic levels can be performed through simulating the drug–receptor interaction process (docking). *In silico* analysis can predict the potency of a drug or a compound of a drug required to produce a particular response. 1 2 The interaction between receptor and glycosaminoglycan-acemannan components can be seen from the presence of cavities; there were specific binding with amino acids of the protein receptor. 12

This *in silico* study aimed to predict osteogenesis activity of properties of glycosaminoglycan-acemannan and its combination as a scaffold material in alveolar bone healing. It shows descriptions of 2D and 3D ligand interaction CAS-LYS-LEU-ARG-LYS-ILE-MSE [A] (Code PDB: 1FYW), glycosaminoglycan-acemannan with amino acids on TLR-2 receptors through hydrogen bonds and strong steric interactions. It shows that there are chemical bonding and interactions of ligands, glycosaminoglycan-acemannan with amino acids on TLR-2 receptors. It is strong predictor of potent osteogenesis activity of glycosaminoglycan-acemannan due to its chemical structure capable of binding to TLR-2 receptor with strong steric interaction. The results of the ligand docking process and the TLR-2 receptor showed that there were five cavities where glycosaminoglycan-acemannan combination compounds were bound. This shows that the combination of glycosaminoglycan-acemannan has good biological activity and is able to bind to the TLR-2 as a target receptor.

Alveolar bone damage releases endogenous DAMP molecules that are recognized by the TLR-2 receptor-regulated signal complex to activate macrophages. Macrophage cells are important inflammatory cells that play a role in the release of important growth factors that support the alveolar bone healing. 4 15 The binding of lectin protein (aloktin) with acemannan polysaccharides will activate the complement system and platelets as well as several blood clotting factors to fill the tooth socket in order of stimulating the migration of macrophage cells. It inactivates nuclear factor kappa-β and causes suppression of levels of inflammatory interleukin (IL)-1, IL-2 cytokine secretion and tumor necrosis factor-α (TNF-α) expression which regulates differentiation of osteoclasts. Macrophages stimulate the release of BMP-2 and VEGF that stimulate osteoblast formation. 13 14 16 17 The glycosaminoglycans promote osteoblast formation, suppresses TNF-α that plays a role in OPG/RANKL/RANK upregulation. 18 The porous scaffold structure will stimulate new cell's growth, osteoblasts, and vascularity that can support the bone healing process. 19 20 Modified chitosan scaffolds contain glycosaminoglycan compound potential for future clinical use in alveolar bone healing. 21 The *in vivo* study of application scaffold contain glycosaminoglycans and acemannan compound can increase VEGF expression and woven alveolar bone healing. 9 22 The result of this *in silico* analysis is consistent with the *in vivo* study.

Docking TLR-2 receptor interaction was conducted by MVD program. Three dimensional images of interaction ligand, glycosaminoglycan-acemannan in the fifth cavity were connected. The glycosaminoglycan-acemannan components are bound to the TLR-2 receptor in all cavities, namely cavity 1, 2, 3, 4, and 5. Cavity 1 has the most stable binding compared with the others. The alignment of the molecules in which atoms of the compound belong to the same ligand in the receptor was performed. It automatically docking and measured the energy values or Moldock score. 3 The amount of interaction energy required to interact with the TLR-2 receptor on the CAS-LYS-LEU-ARG-LYS-ILE-MSE [A] ligand is almost the same as the energy of acemannan compound or lesser when compared with glycosaminoglycan compound. This result shows that the acemannan interaction with the TLR-2 receptor is more stable and has better activity than glycosaminoglycans. The smaller the energy required for the ligand and receptor to interact, the more stable the interaction and biological activity of these compounds. 1 2 3 The *in silico* analysis supported the predictive osteogenesis activity of the glycosaminoglycan and acemannan compound on the TLR2 receptor. This result suggests that the combination glycosaminoglycan and acemannan compound has potential as a scaffold material in alveolar bone healing.

## Conclusion

The compound of glycosaminoglycan-acemannan as a scaffold material is able to bind to a TLR-2 target receptor, which predicted osteogenesis activity in alveolar bone healing supported by *in silico* analysis.
